# Qualitative identification of growth hormone-releasing hormones in human plasma by means of immunoaffinity purification and LC-HRMS/MS

**DOI:** 10.1007/s00216-016-9377-3

**Published:** 2016-02-15

**Authors:** Andre Knoop, Andreas Thomas, Eric Fichant, Philippe Delahaut, Wilhelm Schänzer, Mario Thevis

**Affiliations:** Institute for Biochemistry-Center for Preventive Doping Research, German Sport University Cologne, Am Sportpark Müngersdorf 6, 50933 Cologne, Germany; Laboratoire d’Hormonologie, C.E.R. Groupe-Département Santé, Rue du Point du jour 8, 6900 Marloie, Belgium; European Monitoring Center for Emerging Doping Agents (EuMoCEDA), Cologne/Bonn, Germany

**Keywords:** LC-HRMS/MS, GHRH, Immunoaffinity, Doping, MSIA™-Tips

## Abstract

The use of growth hormone-releasing hormones (GHRHs) is prohibited in sports according to the regulations of the World Anti-Doping Agency (WADA). The aim of the present study was to develop a method for the simultaneous detection of four different GHRHs and respective metabolites from human plasma by means of immunoaffinity purification and subsequent nano-ultrahigh performance liquid chromatography-high resolution/high accuracy (tandem) mass spectrometry. The target analytes included Geref (Sermorelin), CJC-1293, CJC-1295, and Egrifta (Tesamorelin) as well as two metabolites of Geref and CJC-1293, which were captured from plasma samples using a polyclonal GHRH antibody in concert with protein A/G monolithic MSIA™ D.A.R.T.’S® (Disposable Automation Research Tips) prior to separation and detection. The method was fully validated and found to be fit for purpose considering the parameters specificity, linearity, recovery (19–37 %), lower limit of detection (<50 pg/mL), imprecision (<20 %), and ion suppression/enhancement effects. The analytes’ stability and metabolism were elucidated using in vitro and in vivo approaches. EDTA blood samples were collected from rats 2, 4, and 8 h after intravenous administration of GHRH (one compound per test animal). All intact substances were detected for at least 4 h but no anticipated metabolite was confirmed in laboratory rodents’ samples; conversely, a Geref metabolite (GHRH_3-29_) was found in a human plasma sample collected after subcutaneous injection of the drug to a healthy male volunteer. The obtained results demonstrate that GHRHs are successfully detected in plasma using an immunoaffinity-mass spectrometry-based method, which can be applied to sports drug testing samples. Further studies are however required and warranted to account for potential species-related differences in metabolism and elimination of the target analytes

**Graphical abstract:**
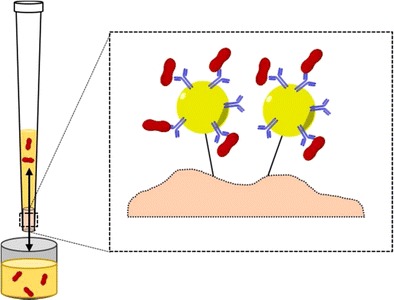
GHRH immunoaffinity purification by means of anti-GHRH antibody coated protein A/G D.A.R.T.’S

GHRH immunoaffinity purification by means of anti-GHRH antibody coated protein A/G D.A.R.T.’S

## Introduction

Growth hormone-releasing hormones (GHRHs) are hypothalamic peptides stimulating the endogenous growth hormone (GH) secretion which, in turn, can entail anabolic effects [1]. Due to their presumed performance enhancing effects for athletes, the World Anti-Doping Agency (WADA) has banned GH and its releasing factors such as GHRH and analogues from sports [2]. As early as 1984, Ling et al. [3] recognized that the human growth hormone-releasing factor (hGRF_1-44_ amide) can be degraded to hGRF_1-29_ amide (Sermorelin) without decreasing its bioactivity to less than 50 %. Various recombinant and synthetic analogues with reduced and also modified amino acid sequences for better metabolic resistance have been investigated, verifying their endocrine effects [4–11]. These studies focused, among other aspects, on identifying metabolic reactions and degradation processes of the truncated and modified analogues of GHRH and specifically determined the protective properties of the introduced D-amino acid moieties against unspecific degradations of the therapeutic peptides in plasma [7]. Further, different confiscated products have corroborated suspicions of a growing GHRH black market and illicit synthesis of analogue peptide hormones. For instance, CJC-1295 ((D-Ala^2^, Gln^8^, Ala^15^, Leu^27^)-GRF amide), an emerging drug candidate, was identified in 2010 [12], and in 2014 the finding of a modified analogue of human growth hormone-releasing hormone (hGHRH_1–44_) was reported [13].

For sports drug testing purposes, the prediction and characterization of the metabolic behavior of particularly peptide-derived substances as well as analytical approaches allowing for appropriately sensitive detection methods are vital. Therefore, four different peptidic compounds with GH-releasing properties exhibiting molecular masses between 3355 and 5132 kDa were intravenously administered to rats, and blood and plasma samples were collected. In addition, a healthy male volunteer received a single subcutaneous dose of Sermorelin and EDTA blood specimens were sampled. All specimens were subjected to established principles of immunoaffinity purification [14–16] followed by nano-ultra high performance liquid chromatography (UHPLC) coupled to high resolution tandem mass spectrometry (HRMS/MS) [17–20]. However, in the present approach, an alternative methodology utilizing an immunoaffinity extraction protocol based on pipette tips containing covalently immobilized recombinant Protein A/G [21] combined with a polyclonal anti-GHRH antibody was employed. Here, besides the aforementioned intact peptidic substances, two synthetic metabolites (degradation products) were included into the test method. For Sermorelin, an in vitro-derived metabolite has been described as originating from the loss of the N-terminal Tyr-Ala-residue due to cleavage by dipeptidyl peptidase-IV (DPP-IV) [7]. For CJC-1293, it was anticipated that N-terminal Tyr-residue will also be enzymatically removed [7, 22].

## Material and methods

### Chemicals and materials

For all aqueous buffers and diluting steps, ultrapure water obtained from a MilliQ device was used. Glacial acetic acid was purchased from Merck (Darmstadt, Germany). Phosphate-buffered saline (PBS, pH 7.4) tablets were obtained from Sigma (Schnelldorf, Germany) and used for preparation of PBS buffer according to the manufacturer’s recommendations (one tablet dissolved in 200 mL of water). The Finnpipette™ Novus i Multichannel Electronic Pipette (including adjustable pipetter stand) and the appropriate Protein A/G MSIA™ D.A.R.T.’S® (96 tips) were obtained from Thermo (San Diego, CA, USA). Corresponding 96-well plates (0.5 mL, polypropylene) were purchased from Agilent Technologies (Waldbronn, Germany). GHRH primary antibody (polyclonal; host, rabbit) was kindly provided from C.E.R.-Groupe Laboratories (Marloie, Belgium). Commercially available pooled human plasma (Octaplas, for intravenous infusion) was purchased from Octapharma (Langenfeld, Germany). The internal standard (ISTD) acetyl-(Tyr^1^, D-Arg^2^)-GRF_1-29_ amide and Tesamorelin (Egrifta™) were obtained from Bachem (Bubendorf, Switzerland). GHRH_1-29_ (hGRF, Geref, Sermorelin) was purchased from the Biologisch-Medizinisches Forschungszentrums (Düsseldorf, Germany). CJC-1295 ((D-Ala^2^, Gln^8^, Ala^15^, Leu^27^)-GRF amide) and CJC-1293 ((D-Ala^2^)-GRF amide) were custom-synthesized by Centic Biotec (Jena, Germany), and the metabolites of Sermorelin (GRF_3-29_) and CJC-1293 ((D-Ala^2^)-GRF_2-29_ amide) were by the Medizinisch-Naturwissenschaftliches Forschungszentrum (Eberhard Karls University, Tübingen, Germany).

### Liquid chromatography (LC)

LC separation was accomplished on an AQUITY nanoflow UHPLC system from Waters (Milford, CT, USA) equipped with a Waters Symmetry trapping column (C18, 180 μm × 20 mm, 5 μm particle size) and a New Objective PicoChip Reprosil pur (C18, 75 μm, 100 mm, 3 μm particle size) as analytical column and emitter. For gradient elution, 0.1 % formic acid was used as solvent A and acetonitrile acidified with 0.1 % formic acid as solvent B. The trapping of 1 μL of sample solution was performed using 1 % B for 3 min with a flow of 8 μL/min. The gradient program for the analytical run, conducted at a flow rate of 350 nL/min, started with 1 % B, increasing to 60 % B within 20 min and to 80 % B in 2 min. Subsequent re-equilibration at 1 % B was accomplished within 8 min.

### Mass spectrometry

For the mass spectrometric detection, a Q Exactive (Thermo, Bremen, Germany) operating in positive ion mode equipped with a nano electrospray ionization source (+ESI) was employed. Ionization was accomplished at a transfer capillary temperature of 150 °C and a spray voltage of 2 kV. Data was generated in full scan mode (*m/z* 400–1500, resolution 35,000 full width at half maximum (FWHM)) and targeted selected ion monitoring (tSIM, resolution 17,500 FWHM), which were multiplexed four times and combined with an inclusion list containing accurate *m/z* values of most abundant charge states of all target peptides. The isolation window of the quadrupole was set to 3 *m/z* for the tSIM and 1.5 *m/z* for data-dependent tandem mass spectrometry (ddMS^2^) experiments, respectively. Accurate mass measurements (<5 ppm) were ensured through calibration according to manufacturer’s recommendations (calibration mixture of caffeine, tetrapeptide MRFA, and Ultramark). The gas supply consisted of nitrogen produced by a nitrogen generator (CMC Instruments GmbH, Eschborn, Germany). Main mass spectrometric parameters and data of the target analytes are shown in Table 1.

**Table 1:** Amino acid sequences and mass spectrometric parameters of target analytes

Peptide	Amino acid sequence	Monoisotopic mass [Da]	Precursor [*m/z*]	Predominant charge state	Retention time [min]
Sermorelin	YADAIFTNSYRKVLGQLSARKLLQDIMSR-NH_2_	3355.8	672	5+	19.6
Sermorelin metabolite	DAIFTNSYRKVLGQLSARKLLQDIMSR-NH_2_	3121.8	625	5+	19.5
CJC-1293	Y**dA**DAIFTNSYRKVLGQLSARKLLQDIMSR-NH_2_	3355.8	672	5+	19.6
CJC-1293 metabolite	**dA**DAIFTNSYRKVLGQLSARKLLQDIMSR-NH_2_	3192.8	639	5+	19.4
CJC-1295	Y**dA**DAIFT**Q**SYRKVL**A**QLSARKLLQDI**L**SR-NH_2_	3365.9	674	5+	19.8
Tesamorelin	**hexenoyl-**YADAIFTNSYRKVLGQLSARKLLQDIMSRQQGESNQERGARARL-NH_2_	5132.7	734	7+	19.7
ISTD	**acetyl-**Y**R**DAIFTNSYRKVLGQLSARKLLQDIMSR-NH_2_	3482.9	697	5+	19.6

### Sample preparation and purification-MSIA™-Tips

**Fig. 1:**
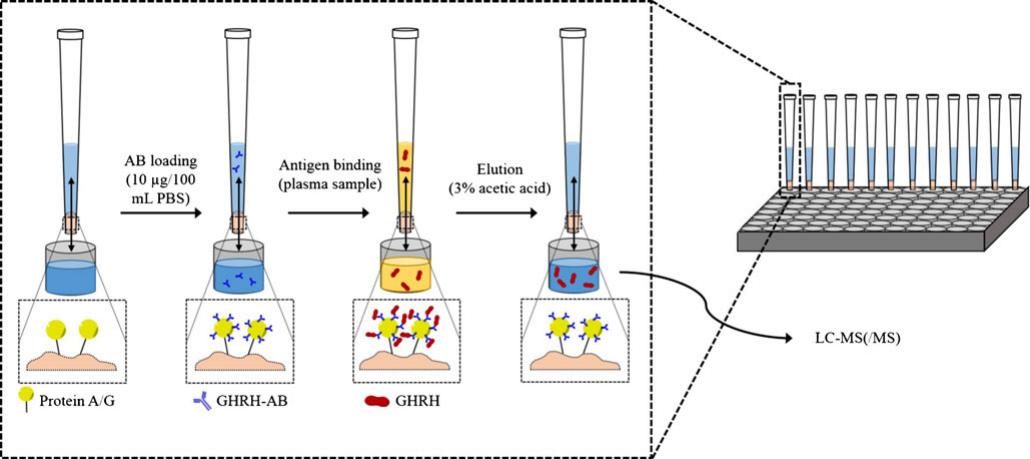
Sample preparation procedure and main steps of MSIA™ purification using protein A/G tips

Sample aliquots of 100 μL of plasma were spiked with 1 μL of ISTD [200 ng/mL] and diluted with 100 μL of PBS before extraction. The multichannel pipette was equipped with up to 12 protein A/G MSIA™-Tips and up to 2000 cycles (one cycle corresponding to one round of liquid aspiration and ejection) were used for distinct sample preparation steps as outlined in Table 2. First, tips were conditioned with PBS before binding 10 μg of the polyclonal GHRH primary antibody (5 μL [2 mg/mL] in 95 μL PBS) to the proteins A/G immobilized on the monolithic adsorbent surface. After washing with PBS to remove unbound antibody residues the target analytes were extracted from plasma by antibody-antigen complex formation prior to three consecutive washing steps with PBS and water (Table 2). The peptides of interest were eluted into LoBind tubes using 3 % of aqueous acetic acid and the eluate was immediately used for injection into the UHPLC-MS(/MS) system. In Fig. 1, a schematic illustration of the relevant principle steps of the MSIA™ purification is presented.

**Table 2:** Pipetting scheme for GHRH purification using a Thermo Finnpipette™ Novus i Multichannel Electronic Pipette equipped with Protein A/G MSIA™-Tips

Step	1	2	3	4	5	6	7	8
	Wash	Antibody load	Wash	Antigen binding	Wash	Wash	Wash	Elution
Liquid	PBS	AB dissolved in PBS [10 μg/mL]	PBS	Plasma sample	PBS	PBS	Water	3 % acetic acid
Cycles	10	999	10	2 × 999	10	10	10	200
Cycle volume [μL]	150	75	150	150	150	150	150	75
Total volume [μL]	300	100	300	200	300	300	300	100

### Qualitative method validation

For qualitative result interpretation, the developed method was validated for human plasma regarding the following parameters: specificity, linearity, recovery, lower limit of detection (LLOD), imprecision, and ion suppression/enhancement effects.

In order to identify potential interfering signals and mean background noise, ten blank plasma specimens (five female and five male) from healthy volunteers without any known medications were prepared as described above to ensure specificity of signals at expected retention times (*n* = 5 + 5). For the evaluation of the assay’s linearity, a mixture containing reference standards of all target analytes was spiked at six concentration levels (0, 0.1, 0.2, 0.4, 1, 2, and 5 ng/mL of each analyte) into blank plasma specimens, which were analyzed as outlined above. The extraction recovery was determined by using 12 plasma aliquots containing 1 ng/mL per analyte. The first set of six samples was fortified before immunoaffinity purification and the other six were spiked after workup and immediately before LC-MS/MS analysis (*n* = 6 + 6). The LLOD was evaluated as the lowest content measurable via signal-to-noise ratio (S/N) of >3 and therefore six biological replicates were prepared and analyzed at the estimated LLOD of 50 pg/mL. To calculate the method’s imprecision, six sample replicates at three different concentrations (0.05, 1, and 5 ng/mL) were analyzed (*n* = 3 × 6) as before. For reasons of matrix effects and influences on ionization (ion suppression or enhancement), peak areas of blank plasma aliquots spiked after sample workup [0.5 ng/mL per analyte] were compared to those of an analogously fortified neat solution (3 % acetic acid).

### In vitro experiments

For the investigation of the metabolic fate of GHRHs, in vitro experiments using fresh human plasma samples were conducted, allowing to test for potential degradation products of the studied substances. In accordance to earlier reported methods [23], aliquots of human plasma samples were diluted (1:1) with PBS [22] and spiked to 10 μg/mL with Sermorelin or Tesamorelin, respectively. After incubation for 30, 60, 120, and 240 min at 37 °C, samples were prepared and analyzed as described elsewhere [20]. Simulating degradation processes of exopeptidases, potential metabolites were considered by eliminating up to three amino acids from both *N*- and *C*-terminus. All samples were probed for the anticipated metabolic products as well as for the corresponding intact drugs.

### Administration studies

#### Rat administration studies

To probe for the in vivo metabolism of Sermorelin, CJC-1293, CJC-1295, and Tesamorelin, 0.1 mg of each GHRH was administered intravenously to three rats (*Rattus norvegicus*, Wistar, 200–300 g bodyweight at dosing). Urine samples were collected in a metabolic cage prior to and 6, 12, 24, and 30 h after administration but not subjected to analysis in this study. Blood was sampled at 2, 4, and 8 h after injection and specimens were centrifuged at 13,000×*g* for 20 min to produce serum or EDTA plasma, which was stored at −20 °C until analysis. The animal studies were conducted with approval of the respective ethical committee and the laboratory followed the directive 2010/63/EU of the European parliament and the council of 22 September 2010 on the protection of animals used for scientific purposes.

#### Human urine and plasma samples

Complementary to the aforementioned rat elimination study, a single dose of 500 μg of Geref (Sermorelin) (Hallandale Pharmacy, FL, USA) was self-administered subcutaneously by a healthy male volunteer (59 years, 78 kg) following the provision of written consent. EDTA plasma samples were collected 30, 90, and 270 min after dosing and the specimens were stored at −20 °C until analysis.

## Results and discussion

All of the herein investigated peptides represent substances that are prohibited at all times according to regulations established by the WADA [2]. To date, information concerning their metabolism and potential windows of detection in a doping control context has been scarce; however, it is of utmost importance to allow for estimating the fitness for purpose of currently employed sports drug testing methods.

### Qualitative method validation

**Fig. 2:**
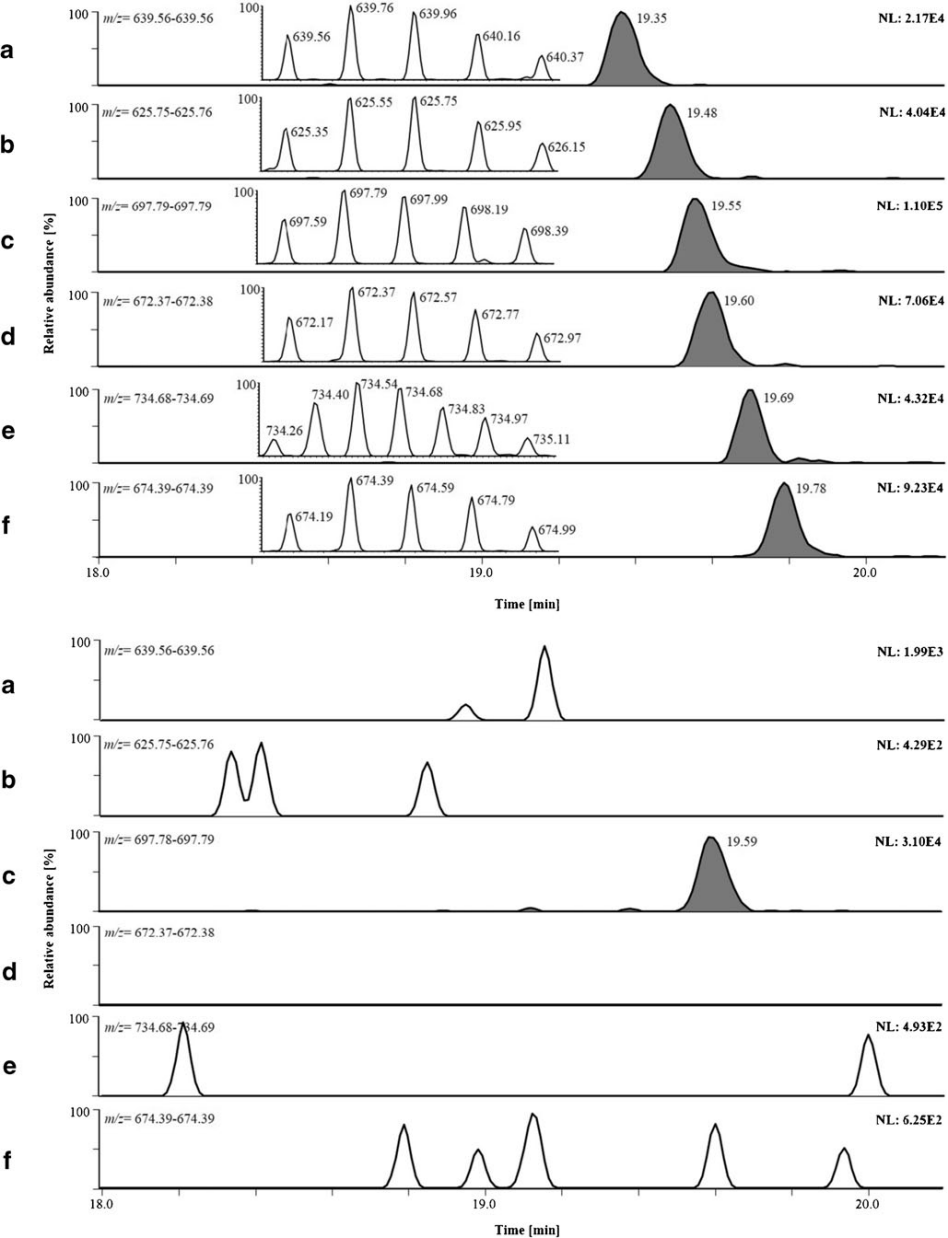
Extracted ion chromatograms (targeted SIM experiments) of [*a*] a metabolite of CJC-1293, [*b*] a metabolite of Geref/Sermorelin, [*c*] the ISTD, [*d*] Geref/Sermorelin and CJC-1293, [*e*] Tesamorelin, and [*f*] CJC-1295, all in human plasma. Corresponding high resolution mass spectra of the protonated molecules [M+5H]^5+^ and [M+7H]^7+^, respectively, are shown as *insets*. A spiked sample at the estimated LLOD of 50 pg/mL is shown as *top panel* and a corresponding blank plasma sample spiked with the ISTD only is presented *below*

Main test method validation results for qualitative analytical purposes are summarized in Table 3. All target peptides were detected as 5-fold or 7-fold charged molecules and corresponding ions were observed at *m/z* 672 for Sermorelin and CJC-1293 (*z* = 5), at *m/z* 674 for CJC-1295 (*z* = 5), at *m/z* 697 for the ISTD (*z* = 5), and at *m/z* 734 for Tesamorelin (*z* = 7) as well as *m/z* 625 and 639 for the metabolites of Sermorelin (*z* = 5) and CJC-1293 (*z* = 5), respectively. The retention times of the analytes varied from 19.35 to 19.78 as depicted in Figs. 2 and 4. The specificity of the assay was demonstrated by the absence of interfering signals in defined extracted ion chromatograms of the analytes at expected retention times. Within the employed working range (0.1–5 ng/mL), the signal responses of all analytes proved linear with coefficients of correlation greater or equal to 0.994. The sample preparation protocol as well as analytical setup were found to be compatible also with serum samples as assessed with a separate calibration curve experiment yielding coefficients of correlation ≥0.978. Analyte recoveries concerning immunoaffinity purification were tested and varied between 19 and 37 %. The estimated LLOD of 50 pg/mL was corroborated with appropriate signal intensities and signal-to-noise ratios substantially greater than 3 for all target compounds as illustrated in Fig. 2. The imprecision of the method was determined as the relative standard deviation of the procedure for low [50 pg/mL], medium [1 ng/mL], and high [5 ng/mL] concentrations ranging from 3.5 to 19.2 %. Comparative spiking experiments between blank plasma samples and neat elution buffer for matrix effects showed no ion suppressing effects [24–26]. On the contrary, higher signal intensities were observed for spiked plasma samples, an effect that was attributed to reduced unspecific losses of the analytes (e.g., on instrument or vial surfaces) in the presence of co-extracted plasma proteins.

**Table 3:** Main validation results for qualitative analysis of GHRHs in human plasma

Parameter		Sermorelin / CJC-1293	CJC-1295	Tesamorelin	Sermorelin metabolite	CJC-1293 metabolite
LLOD [pg/mL]		<50	<50	<50	<50	<50
Slope		13.157	13.099	6.735	6.432	8.000
Intercept		−1.091	−1.292	−0.506	−0.378	−1.063
Coefficient of correlation		0.998	0.995	0.994	0.998	0.998
Imprecision [%]	50 pg/mL	5.2	14.5	16.4	16.2	7.9
	1 ng/mL	7.6	5.4	3.5	8.4	6.8
	5 ng/mL	10.0	13.5	19.2	10.4	14.3
Recovery [%]		23	19	37	27	30

### In vitro experiments

Identifying potential metabolites of Sermorelin and Tesamorelin was accomplished by subtracting and comparing mass spectrometric full scan data of samples prepared before and after incubation of the substrates in fresh human plasma. For Tesamorelin, no degradation products of considerable abundance were detected, suggesting a substantial resistance towards proteolytic activities in human plasma. Conversely, in case of Sermorelin, the earlier reported metabolite (GRF_3-29_) [7] was observed as a result of in vitro metabolic processes and the amounts of the intact drug decreased continuously with an increasing duration of incubation. After a period of 4 h, Sermorelin was entirely degraded from the spiked plasma sample.

### In vivo experiments

**Fig. 3:**
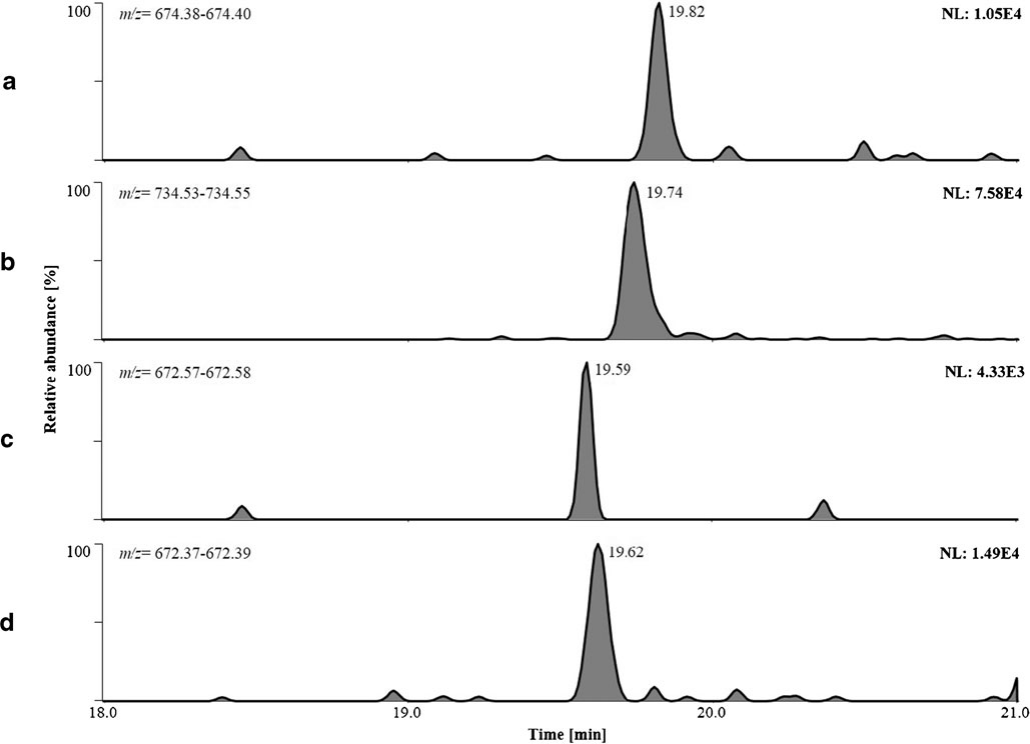
Extracted ion chromatograms (targeted SIM experiments) of [*A*] CJC-1295 (8 h post-administration), [*B*] Tesamorelin (8 h post-administration), [*C*] CJC-1293 (8 h post-administration), and [*D*] Geref/Sermorelin (2 h post-administration) in rat plasma after intravenous injection of 0.1 mg of the drug

All intact GHRHs administered intravenously to rats were detected in plasma samples collected 2 h post dosing. In addition, Tesamorelin, CJC-1295, and CJC-1293 were also identified in specimens sampled 4 and 8 h post-administrating (Fig. 3), with the 8-h sample representing the latest collection time point of the study. Here it appears noteworthy that only qualitative results were obtained and that no pharmacokinetic interpretation was conducted. While all analytes were unequivocally identified and differentiated by mass spectrometry, chromatographic separation was accomplished for all target peptides with the exemption of the closely related compounds Sermorelin and CJC-1293 under the chosen analytical conditions. Both Sermorelin and CJC-1293, which differ only by the substitution of *l*-alanine by *d*-alanine at position 2 (Table 1), were found to be readily biotransformed especially when compared to CJC-1295. However, none of the anticipated metabolic products such as N- or C-terminally truncated analogues were detected, suggesting the rapid and unspecific degradation of these compounds.

**Fig. 4:**
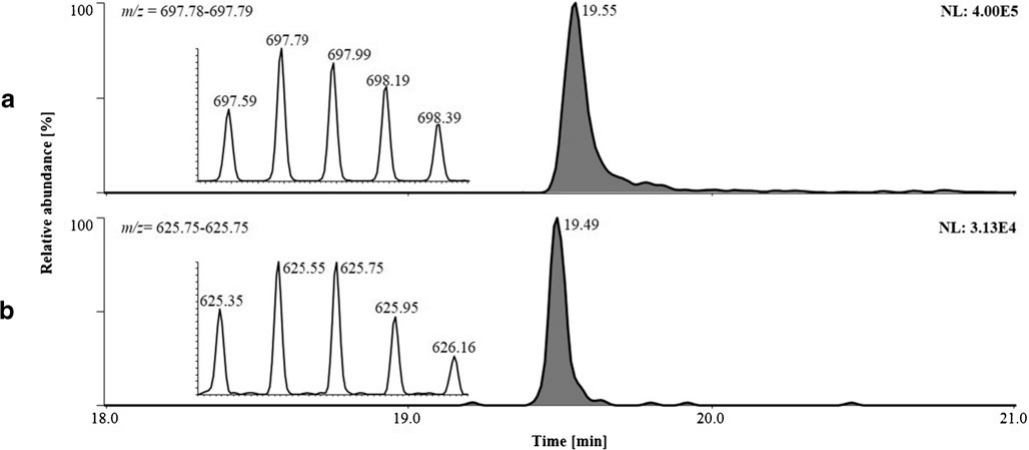
Extracted ion chromatograms (targeted SIM experiments) of [*A*] the ISTD and [*B*] a Geref/Sermorelin metabolite in human plasma 30 min after subcutaneous application of 500 μg of Geref/Sermorelin to a healthy male volunteer. Corresponding high-resolution mass spectra of the protonated molecules [M+5H]^5+^ are shown as *insets*

Compared to rat plasma samples collected after the intravenous application of Sermorelin, the human in vivo study (using subcutaneous drug application) indicates a different but also rapid metabolism of the peptide hormone. While the intact drug was not detected in any of the collected plasma samples, the in vitro-derived Sermorelin metabolite GRF_3-29_ was detected in plasma 30 min post injection (Fig. 4). The subsequently collected specimen sampled after 90 min returned negative test results for the selected metabolite, suggesting the need for further metabolic products to allow for extended detection windows of the prohibited peptide hormone. In addition to EDTA plasma, also serum samples were collected at identical time points yielding comparable results (data not shown), which corroborated the possibility of using both matrices (i.e., serum and plasma) for doping control purposes.

In accordance to former GHRH pharmacokinetic studies by Jetté et al. [7], comparable metabolic stabilities of Sermorelin, CJC-1293, and CJC-1295 were observed. The results of the present assay confirm that Sermorelin is effectively metabolized and the GRF_3-29_ metabolite was found instead of the active substance in case of subcutaneous administration (to humans) as well as in vitro. Because of their D-Ala^2^ modification, no analogous metabolites of CJC-1293 and CJC-1295 were observed. Here, the intact compounds were detectable up to the latest sample collection time point and proved significantly longer-lasting, suggesting to include the administered substance in doping control analytical approaches. Similarly, the modified GHRH analogue Tesamorelin demonstrated considerable metabolic stability that enabled its identification at all sampling points (i.e., up to 8 h post-administration).

## Conclusion

GHRHs are prohibited in elite sports at all times and, consequently, test methods are required that enable WADA-accredited laboratories to detect these analytes or respective metabolic products in routine doping control samples. In the light of the limited amount of information regarding the in vivo degradation of the GHRH-derived compounds Sermorelin, CJC-1293, CJC-1295, and Tesamorelin, both in vitro simulation as well as animal and human in vivo studies were conducted allowing to identify practical target analytes (i.e., intact compounds and/or diagnostic metabolites) to estimate detection windows and to establish and validate a test method utilizing a semi-automatable immunoaffinity purification (Protein A/G MSIA™ D.A.R.T.’S®) approach followed by UHPLC-HR-MS(/MS) analysis. The developed assay was found to be fit for purpose, and the intact compounds and/or metabolites were detected for up to 8 h in administration study plasma samples. In case of Sermorelin, the detection window was limited to 2 h following a single dose application. These results suggest follow-up studies potentially enabling the identification of additional metabolites offering extended detection windows.
